# Dual nonlinearity Controlling of Mode and Dispersion Properties in Graphene-Dielectric Plasmonic Waveguide

**DOI:** 10.1186/s11671-017-2166-x

**Published:** 2017-06-08

**Authors:** Xiangqian Jiang, Jinlin Bao, Bing Zhang, Xiudong Sun

**Affiliations:** 1 Department of Physics, Harbin Institute of Technology, Harbin, 150001 China; 2 Collaborative Innovation Center of Extreme Optics, Shanxi University, Taiyuan, 030006 China

**Keywords:** Dual nonlinearity, Graphene, Plasmonic waveguide, Dispersion, 78.67.Wj, 42.65.Wi

## Abstract

We study the mode and dispersion properties of graphene-dielectric nonlinear plasmonic waveguide considering the dual nonlinearity of dielectric and graphene. For TM polarization, the mode distribution, the permittivity distribution, and dispersion relation were obtained by numerically solving the Maxwell equations. Compared with the case considering only the nonlinearity of dielectric, the initial field intensity to excite plasmon modes reduces obviously when introducing the dual nonlinearity. In addition, the influence of dual nonlinearity on dispersion relation is discussed, and we find that the graphene’s nonlinearity affects strongly the dispersion properties. The introduction of dual nonlinearity leads to the decrease of the initial field intensity, which has potential application in all-optical switches with low threshold.

## Background

The graphene plasmonics have attracted widespread attention [1–4] due to the unique electronic and optical features of graphene compared with metals. At THz and far-infrared frequency range, the intraband transition of electrons dominates and graphene behaves like a metal. Therefore, the surface plasmon polaritons (SPPs) could be supported by graphene. For the graphene-dielectric multilayer composite structure, the modes excitation, coupling, and propagation of SPPs have been investigated. The quasi-transverse electromagnetic mode was found in a graphene parallel-plate waveguide [5]. The coupling of SPPs were studied [6, 7] in a graphene-dielectric multilayer structure. For the monolayer graphene sheet periodic array structure, strong coupling between SPPs emerges when the graphene sheets are arranged tightly.

Considerable efforts have been devoted to investigating the optical properties of graphene-dielectric nonlinear composite structures [8–12] for their great potential in controlling light propagation at the micro-and nano-scales. For the single layer graphene case, the surface plasmons at the interface between graphene and kerr-type nonlinear substrate were discussed [8]. It is shown that the wavelength of graphene plasmons can be tuned by adjusting the nonlinear permittivity of substrates. For the graphene-nonlinear dielectric multilayer structure, the propagation and localization properties of graphene plasmons were explored, and the exact dispersion relations for TM surface plasmons of a graphene parallel plate waveguide were obtained [11]. The propagation and localization length are remarkably affected by adjusting nonlinear permittivities. Recently, the dispersion relation for the symmetric and antisymmetric plasmon modes has been derived in a graphene-coated kerr slab structure [12]. Except for the typical forward-propagating mode, the symmetric, and antisymmetric modes were found.

Based on the graphene’s strong nonlinearity, several nonlinear optical effects have been predicted [13–17]. Nesterov et al. [15] studied the nonlinear propagation of light in a graphene monolayer, and found that graphene monolayer can supports TE and TM spatial optical soliton at optical frequencies due to the intrinsic nonlinearity of graphene. More recently, replacing monolayer graphene by multilayer graphene, Smirnova et al. [16] investigated the nonlinear properties of a multilayer stack of graphene sheets, and derived the nonlinear equations describing spatial dynamics of the nonlinear plasmons. The previous studies mainly focused on the influence of single nonlinearity on control of light properties in graphene-dielectric structures. The idea of dual nonlinearity control was introduced in the graphene-based photonic superlattices [18, 19], in which the electrical and all-optical control of photonic beams with deep-subwavelength accuracy was achieved. However, the dual nonlinearity control of mode and dispersion properties in graphene-dielectric plasmonic structure still leaves open many questions. Therefore, in this paper we consider the graphene’s and dielectric’s nonlinearity simultaneously in the graphene-dielectric-graphene waveguide, and study the influence of the dual nonlinearity on modes coupling and dispersion properties.

## Methods

**Fig. 1:**
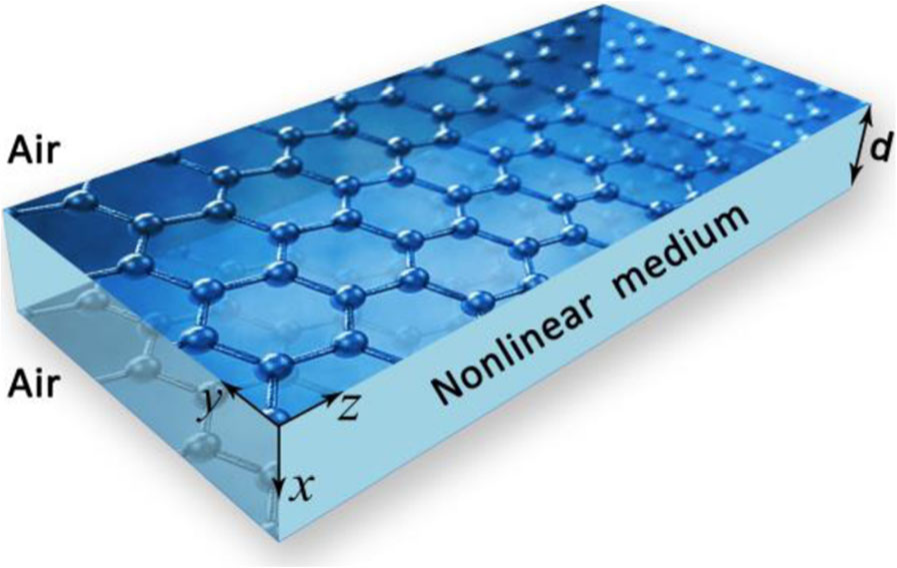
Schematic diagram of nonlinear graphene-dielectric-graphene plasmonic waveguide

The graphene-nonlinear dielectric plasmonic waveguide is schematically illustrated in Fig. 1, a graphene parallel plate with a conductivity *σ*_*g*_ is placed at *x* = ± *d*/2, where the dielectric is a kerr-type medium with a permittivity *ε* = *ε*_*L*_ + *α*|*E*|^2^. In our analysis, the graphene is treated as a boundary due to its thickness in one atom scale. Considering a transverse-magnetic(TM) SPPs that propagate along *z* direction with a propagation constant *β* and exponentially decays along the *x* direction into the air and nonlinear medium, respectively.

For the TM polarization, we know that there are three field components *E*_*x*_, *E*_*z*_, and *H*_*y*_. The magnetic field **H** = *H*_*y*_ **y** and electric field **E** = *E*_*x*_ **x** + *E*_*z*_ **z** satisfy the equations

1 $$ \frac{d{E}_z}{ d x}= i\omega {\mu}_0{H}_y+ i\beta {E}_x $$

2 $$ i\beta {H}_y=- i\omega {\varepsilon}_0\varepsilon {E}_x $$

3 $$ \frac{d{ H}_y}{ d x}= i\omega {\varepsilon}_0\varepsilon {E}_z $$

where *ε*_0_ and *μ*_0_ are the electric permittivity and magnetic permeability of vacuum. From Eq. (2) and *ε* = *ε*_*L*_ + *α*|*E*|^2^ we can get

4 $$ {\varepsilon}^2{E}_x^2=\frac{\beta^2}{\omega^2{\varepsilon}_0^2}{H}_y^2 $$

5 $$ {E}_x^2=\left(\varepsilon -{\varepsilon}_L-\alpha {E}_z^2\right)/\alpha $$

Substituting Eq. (5) into Eq. (4) we have

6 $$ {\varepsilon}^3-\left({\varepsilon}_L+\alpha {E}_z^2\right){\varepsilon}^2-\frac{\alpha {\beta}^2}{\omega^2{\varepsilon}_0^2}{H}_y^2=0 $$

For cubic equation [20, 21]

7 $$ {x}^3+ b{x}^2+ c x+ d=0 $$

The discriminant of Eq. (7) is

8 $$ \varDelta ={b}^2{c}^2-4{c}^3-4{b}^3 d+18 b c d-27{d}^2 $$

Setting $b=-\left({\varepsilon}_L+\alpha {E}_z^2\right),\kern0.5em  c=0$, and $d=-\alpha {\beta}^2{H}_y^2/\left({\omega}^2{\varepsilon}_0^2\right)$, it is easy to demonstrate that the discriminant of Eq. (6) meets

9 $$ \varDelta =-{\left({\varepsilon}_L+\alpha {E}_z^2\right)}^3\frac{\alpha {\beta}^2}{\omega^2{\varepsilon}_0^2}{H}_y^2-27\frac{\alpha^2{\beta}^4}{\omega^4{\varepsilon}_0^4}{H}_y^4<0 $$

*Δ* < 0 means that the Eq. (6) only has one real solution. From Cardano’s method [20], we know that for the cubic equation Eq. (7) it’s real root is

10 $$ x=-\frac{b}{3}+\sqrt[3]{-\frac{q}{2}+\sqrt{{\left(\frac{p}{3}\right)}^3+{\left(\frac{q}{2}\right)}^2}}+\sqrt[3]{-\frac{q}{2}-\sqrt{{\left(\frac{p}{3}\right)}^3+{\left(\frac{q}{2}\right)}^2}} $$

where *p* = *c* − *b* ^2^/3, *q* = *d* − *bc*/3 + 2*b* ^3^/27. Using Eq. (10) we can obtain the *ε*. Replacing the *ε* in Eq. (2) and (3) by the real solution, the ordinary differential equations can be solved numerically by a relaxation method.

## Results and Discussions

From continuity requirements of *E*_*z*_ and *H*_*y*_, the boundary conditions at *x* = ± *d*/2 satisfy *E*_1*z*_ = *E*_2*z*_ and *H*_2*y*_ − *H*_1*y*_ = *σ*_*g*_ *E*_*z*_. The surface conductivity of graphene *σ*_*g*_ is governed by the Kubo formula [22] including the interband and intraband transition contributions. In the THz and far-infrared frequency range, the intraband transition contribution dominates and the surface conductivity can be simplified to a simple Drude type as [23]

11 $$ {\sigma}_g=\frac{e^2{\mu}_c}{\pi {\hslash}^2}\frac{\mathrm{i}}{\omega +\mathrm{i}{\tau}^{-1}} $$

where *e* is the electron charge, *μ*_*c*_ is the chemical potential of graphene, *ω* is the frequency, and *τ* is the momentum relaxation time. This model is applicable in low temperature limit (*k*_*B*_ *T* < < *μ*_*c*_) at low frequency (*ℏω* ≤ *μ*_*c*_). For the strong field condition, the nonlinear part of the conductivity must be considered and the total conductivity of graphene reads [16]

12 $$ {\sigma}_g={\sigma}_L+{\sigma}^{NL}{\left|{E}_{\tau}\right|}^2 $$

where *E*_*τ*_ is the tangential component of the electric field and *σ* ^*NL*^ denotes nonlinear conductivity [16]

13 $$ {\sigma}^{NL}=- i\frac{3}{8}\frac{e^2}{\pi {\hslash}^2}{\left(\frac{e{\nu}_F}{\mu_c\omega}\right)}^2\frac{\mu_c}{\omega} $$

where *ν*_*F*_ = 0.95 × 10^8^cm/s is the Fermi velocity.

For the graphene, only in THz and far-infrared frequency range can its surface conductivity be simplified to a simple Drude type; therefore, we choose the incident wavelength as *λ* = 10 *μm*. Other parameters are fixed to the values *ε*_1_ = 1, *ε*_*L*_ = 2.25, *α* = 5 × 10^− 16^(m/v)^2^ [24] *E*_*F*_ = 0.27 ev, *τ* = 1.5 ps. It is well known that there are two modes in graphene-dielectric-graphene linear structures, which are symmetric and antisymmetric modes, respectively. In the following, we will discuss the influence of nonlinearity on modes distribution in the graphene- dielectric composite structures.

**Fig. 2:**
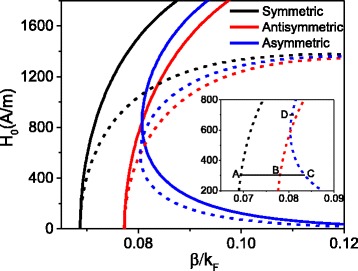
The initial magnetic intensity versus the propagation constant. For the *solid curves*: *α* = 5 × 10^− 16^(*m*/*v*)^2^, *σ* ^*NL*^ = 0; for the *dashed curves*: *α* = 5 × 10^− 16^(*m*/*v*)^2^, *σ* ^*NL*^ = 2.19 × 10^− 20^i, the *horizontal black solid line* is an auxiliary line

**Fig. 3:**
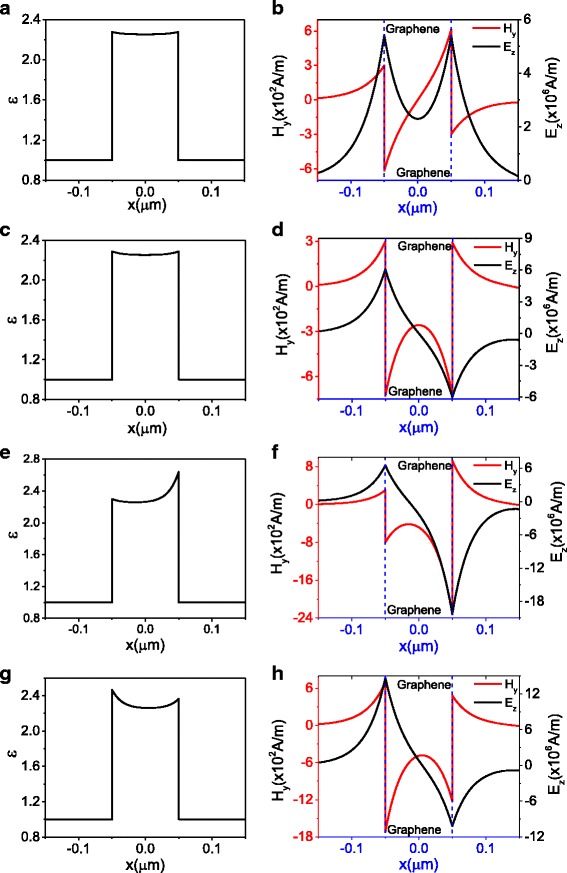
The permittivity and modes distribution for magnetic component *H*_*y*_ and electric component *E*_*z*_. **a** and **b** correspond the point A (*H*_0_ = 300, *β* = 6.94 × 10^− 2^ *k*_*F*_) marked in Fig. 2 for symmetric modes, **c** and **d** correspond the point B (*H*_0_ = 300, *β* = 7.81 × 10^− 2^ *k*_*F*_) marked in Fig.2 for antisymmetric modes, **e** and **f** correspond the point C (*H*_0_ = 300, *β* = 8.36 × 10^− 2^ *k*_*F*_) marked in Fig. 2 for asymmetric modes, and **g** and **h** correspond the point D (*H*_0_ = 700, *β* = 8.07 × 10^− 2^ *k*_*F*_)

Setting *H*_0_ as the initial magnetic field component at incident interface, by solving Eqs.(1, 2, and 3) numerically, the dependence of initial magnetic field intensity *H*_0_ on the propagation constant *β* is given in Fig. 2. The normalized propagation constant ${k}_F=\sqrt{\uppi n}$ is in units of Fermi momentum [25], where *n* = 6 × 10^12^ cm^− 2^ is carrier density. The solid curves represent the case that only the nonlinearity of dielectric is considered, while the dashed curves denote the case that the nonlinearity of dielectric and graphene are considered simultaneously. From Fig. 2 we find that the modes properties for both cases are the same. There are three branches which means the nonlinear plasmonic waveguide can support three modes. However, compared with single nonlinearity case, the initial field intensity reduced apparently for the dual nonlinearity case. Although the graphene nonlinear plasmonic waveguide can support three modes, it is impossible to distinguish which branch denotes symmetric, antisymmetric or asymmetric mode. In order to determine the mode properties of each branch, we plot electric field and magnetic field distribution associated with A, B, C, and D in Fig. 3, respectively.

For the branch of black dashed curve, the corresponding permittivity and fields associated with A are plotted in Fig. 3a, b, in which the distribution of the permittivity and the electric field *E*_*z*_ is symmetric. Therefore, this branch represents the symmetric mode. For the branch of red dashed curve, the permittivity and fields associated with B are given in Fig. 3c, d. Distribution of permittivity is still symmetric; however, the distribution of electric field *E*_*z*_ is antisymmetric which implies this branch is an antisymmetric mode. The distribution of permittivity and field associated with C and D are plotted in Fig. 3e–h. It is noted that the distribution of corresponding magnetic field and electric field associated with C and D is asymmetric; therefore, the branch of blue dashed curve represents asymmetric mode. Meanwhile, the asymmetric distribution of electric field leads to the asymmetric distribution of permittivity.

**Fig. 4:**
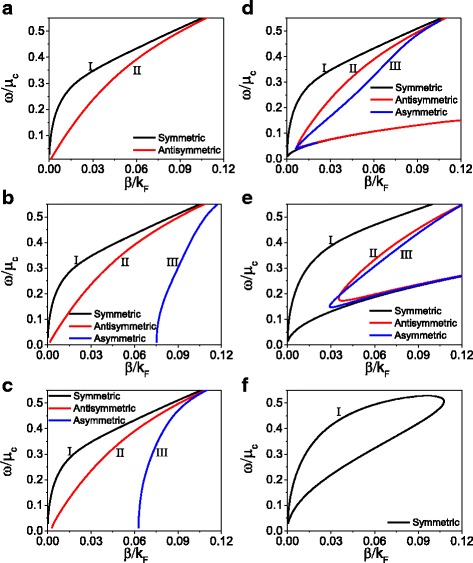
The dispersion relation for a fixed initial magnetic intensity (*H*_0_ = 300 A/m) and for various nonlinear coefficient(**a**–**c**) and for various chemical potential(**d**–**f**). **a** *α* = 0, *μ*_*c*_ = 0.27eV, *σ*_*NL*_ = 0, **b** *α* = 5 × 10^− 17^(m/V)^2^, *μ*_*c*_ = 0.27eV, *σ*_*NL*_ = 0, **c** *α* = 5 × 10^− 16^(m/V)^2^, *μ*_*c*_ = 0.27eV, *σ*_*NL*_ = 0, **d** *μ*_*c*_ = 0.27eV, *α* = 5 × 10^− 16^(m/V)^2^, (**e**) *μ*_*c*_ = 0.16eV, *α* = 5 × 10^− 16^(m/V)^2^, and **f** *μ*_*c*_ = 0.10eV, *α* = 5 × 10^− 16^(m/V)^2^

Next, we turn our attention to discuss the influence of nonlinearity of dielectric and graphene on dispersion relation. Figure 4 shows the dispersion relation for a fixed initial magnetic field (*H*_0_ = 300 A/m) and different chemical potential and nonlinear coefficients of dielectric. In Fig. 4a–c, the influence of nonlinear coefficient of dielectric on dispersion relation is shown, where only the nonlinearity of dielectric is considered. When both the nonlinear coefficient and the nonlinear conductivity equal to zero (*α* = 0, *σ* ^*NL*^ = 0), the nonlinear structure degenerate into a linear structure. In Fig. 4a, for the linear case, only symmetric and antisymmetric modes exist. The black solid curve and the red solid curve represent the symmetric and antisymmetric modes, respectively. When the nonlinear coefficient is nonzero, an asymmetric mode as the branch III shown in Fig. 4b, c appears in the structure. As the nonlinear coefficient increases furtherly, the influence of the coefficient on dispersion properties becomes weak.

In the following, we introduce simultaneously the nonlinearity of dielectric and graphene, and discuss the influence of nonlinearity of graphene on dispersion relation with a fixed nonlinear coefficient of dielectric *α* = 5 × 10^− 16^(m/V)^2^. The results are shown in Fig. 4d-f. Compared Fig. 4d with Fig. 4c, it is noticed that the fold-back phenomenon of dispersion relation appears in all three branches. From Eq. (13), we know that the graphene’s nonlinearity can be controlled by adjusting the chemical potential. As the nonlinearity of graphene further increases from *μ*_*c*_ = 0.27 eV to *μ*_*c*_ = 0.16 eV, as shown in Fig. 4e, the fold-back point of dispersion relation moves up. For a larger nonlinearity of graphene (with small chemical potential *μ*_*c*_ = 0.10eV), as shown in Fig. 4f, only the symmetric mode appears and forms a closed loop. From Fig. 4, we know that considering only the nonlinearity of dielectric, the dispersion relation shows three branches which are almost unchangeable as the nonlinear coefficient of dielectric increases. However, when we further introduce the nonlinearity of graphene, the fold-back phenomenon of dispersion relation appears. For the specified initial magnetic field *H*_0_ and chemical potential the dispersion relation only shows a symmetric mode with a closed loop.

## Conclusions

In summary, we have investigated the mode and dispersion properties of graphene-dielectric nonlinear plasmonic waveguide. The mode distribution, permittivity, and dispersion relations were obtained by numerically solving Maxwell equation for TM polarization. Compared with the case considering only the dielectric’s nonlinearity, the initial field intensity reduced apparently when considering the nonlinearity of dielectric and graphene simultaneously. In addition, the dual nonlinearity affects the dispersion properties of the waveguide significantly. Especially, as the graphene’s nonlinearity increases, the antisymmetric and asymmetric modes merge into one and gradually disappear. Therefore, only the symmetric mode can be found in the case of strong nonlinearity.
